# Prospects of Indole derivatives as methyl transfer inhibitors: antimicrobial resistance managers

**DOI:** 10.1186/s40360-020-00402-9

**Published:** 2020-05-04

**Authors:** Suprim Tha, Sapana Shakya, Rajani Malla, Pramod Aryal

**Affiliations:** Central Department of Biotechnology, Tribhuvan University, Kirtipur, Kathmandu, Nepal

**Keywords:** In silico drug design, Drug resistance, Indoles, SAM, Molecular docking

## Abstract

**Background:**

It is prudent that novel classes of antibiotics be urgently developed to manage the WHO prioritized multi-drug resistant (MDR) pathogens posing an unprecedented medical crisis. Simultaneously, multiple essential proteins have to be targeted to prevent easy resistance development.

**Methods:**

An integration of structure-based virtual screening and ligand-based virtual screening was employed to explore the antimicrobial properties of indole derivatives from a compound database.

**Results:**

Whole-genome sequences of the target pathogens were aligned exploiting DNA alignment potential of MAUVE to identify putative common lead target proteins. S-adenosyl methionine (SAM) biosynthesizing MetK was taken as the lead target and various literature searches revealed that SAM is a critical metabolite. Furthermore, SAM utilizing CobA involved in the B12 biosynthesis pathway, Dam in the regulation of replication and protein expression, and TrmD in methylation of tRNA were also taken as drug targets. The ligand library of 715 indole derivatives chosen based on kinase inhibition potential of indoles was created from which 102 were pursued based on ADME/T scores. Among these, 5 potential inhibitors of MetK in *N. gonorrhoeae* were further expanded to molecular docking studies in MetK proteins of all nine pathogens among which 3 derivatives exhibited inhibition potential. These 3 upon docking in other SAM utilizing enzymes, CobA, Dam, and TrmD gave 2 potential compounds with multiple targets. Further, docking with human MetK homolog also showed probable inhibitory effects however SAM requirements can be replenished from external sources since SAM transporters are present in humans.

**Conclusions:**

We believe these molecules 3-[(4-hydroxyphenyl)methyl]-6-(1H-indol-3-ylmethyl)piperazine-2,5-dione (ZINC04899565) and 1-[(3S)-3-[5-(1H-indol-3-ylmethyl)-1,3,4-oxadiazol-2-yl]pyrrolidin-1-yl]ethanone (ZINC49171024) could be a starting point to help develop broad-spectrum antibiotics against infections caused by *N. gonorrhoeae, A. baumannii, C. coli, K. pneumoniae, E. faecium, H. pylori, P. aeruginosa, S. aureus* and *S. typhi*.

## Background

The finding and development of antibiotics was a milestone in medical sciences that prevented fatality from simple infections. Unfortunately, the emergence of antibiotic-resistant strains among these pathogens appears to be inevitable as selective pressure for survival [1]. The most alarming is the prevalence of resistance even in the last resort antibiotic colistin [2] that has added a serious challenge to the current antibiotic crisis.

The cost of developing a prescription drug estimated by The Tufts Center for the Study of Drug Development as published in the Journal of Health Economics in March 2016, is a massive 2.558 billion dollars [3]. Huge research costs and numerous failures in various stages of drug development have lowered the interests of commercial pharmaceutical companies in drug discovery research. The rapid increase in drug resistance among pathogens and the excessive time and cost parameters required to develop a drug demand a robust and faster method of drug discovery. This is where computational strategies come into play, efficiently assisting drug discovery and development with the available in vitro techniques [4].

Computer-aided drug design (CADD) approaches were applied in this study to find the probable drug targets and discover potential lead candidates against these. SAM is a critical metabolite involved in several biochemical reactions in bacteria. MetK*, a* SAM producer and various SAM utilizers including DNA adenosine methylase (Dam), Uroporphyrinogen-III methyltransferase (CobA), and tRNA (guanine-N (1)-)-methyltransferase (TrmD) were taken as drug targets in this study. Dam is responsible for DNA replication and mRNA transcription which methylates adenine in DNA of bacteria in contrary to human cytosine. TrmD is responsible for proper reading of codons that prevents + 1 frameshift reading error thus involved in proper peptide elongation. CobA is responsible for corrin ring contraction in vitamin B12 synthesis, an important cofactor for membrane biosynthesis. Thus, all the genes/proteins involved from DNA replication to peptide elongation, and even membrane biosynthesis were targeted to discover new lead candidates, simultaneously preventing easy resistance buildup in these targets.

## Methods

### Selection of MDR strains and obtaining their genomic sequences

Nine prioritized pathogens by WHO [5] as ‘critical’ and ‘high’ against whom new antimicrobials are sought were taken as the reference organisms. The whole-genome sequences of these organisms published in NCBI were taken for whole-genome alignment. The genomic sequences of the 9 selected pathogens were downloaded from NCBI FTP site in the annotated gbk format.

ftp://ftp.ncbi.nlm.nih.gov/genomes/archive/old_genbank/Bacteria/

### Multiple sequence alignment (MSA)

MSA was performed using the progressive Mauve algorithm in MAUVE, a multiple sequence alignment software. The genomic regions common to all the aligned sequences were searched for, in MAUVE via visual observation of locally collinear blocks (LCBs) denoted by certain color codes. LCBs represent homologous regions of sequence shared by two or more of the genomes under study without rearrangement [6].

### Alignment of amino acid sequences of the lead proteins

Clustal Omega was used to align the amino acid sequences of S-adenosyl methionine synthase (*MetK*) for all 9 selected pathogens including *Acinetobacter baumannii* strain 1656–2 [7], *Campylobacter coli* 15–537,360 [8], *Enterococcus faecium* Aus0004 [9], *Helicobacter pylori* 2017 [10], *Klebsiella pneumoniae subsp. pneumoniae* 1084 [11], *Neisseria gonorrhoeae* FA 1090 (https://www.ncbi.nlm.nih.gov/nuccore/AE004969) *P. aeruginosa* B136–33 (https://www.ncbi.nlm.nih.gov/nuccore/NC_020912.1) *Staphylococcus aureus* 04–02981 (https://www.ncbi.nlm.nih.gov/nuccore/NC_017340.1) and *Salmonella typhimurium* [12].

### Gene essentiality analysis

The common genes obtained from MAUVE alignment were looked for their essentiality in DEG and OGEE, databases of essential genes.

### Obtaining the dockable crystal structures of the target proteins

The X-ray diffraction structures of S-adenosyl methionine synthase, MetK from *N. gonorrhoeae* (PDB id: 5T8S) [13]; cobA from *P. aeruginosa* (PDB id: 2YBQ) [14] and that of TrmD from *P. aeruginosa* (PDB id: 5WYQ) [15] were obtained from Protein Data Bank. For those whose crystal structures were not available in RCSB PDB, homology modeling tools including Phyre2, RaptorX, ps2v2, Swiss-model, and CPHmodel were used to predict their tertiary structures and the best structures were selected based upon the completeness and Z-scores of the predicted structures using Prosa-server.

### Preparation of ligand database

In the present work, both ligand-based (LBVS) and structure-based virtual screening (SBVS) was performed. LBVS was done because similar compounds exhibit similar Physico-chemical and biological properties so a broad chemical database with structural diversity would offer an ideal solution for effective lead discovery. In this study, a ligand database containing 715 indole derivatives including marine indoles [16] was prepared from ZINC database [17].

### Protein and ligand preparation

SBVS was performed based on the common gene in all nine pathogens, MetK, and the metabolite that it produces, SAM which is further utilized in methylation reactions. Prior to molecular docking, the proteins and ligands were prepared for efficient and more accurate docking results. Protein preparation was done by deleting water, adding hydrogen atoms, merging non-polar bonds, and computing Gasteiger charges in AutoDockTools (http://mgltools.scripps.edu/). Similarly, ligand preparation was done in Openbabel GUI [18] available in PyRx interface by adding hydrogens, energy minimization and converted to pdbqt file format, a useable file format for docking afterward.

### Setting reference values for docking

The native ligands were removed from each of the target proteins in Discovery Studio Visualizer 2017 and docked back in their binding sites, a process called re-docking. The binding energy thus calculated was taken as a reference value for identifying potential leads based on their binding energy in the respective binding pockets of the target proteins.

### Binding sites prediction

The ligand-binding sites in the target proteins were identified from the RCSB protein data bank. For the homology modeled 3D-structures, 3DLigandSite, a web server that superimposes the ligands bound to the structures similar to the query and thus predicts the binding site [19], was used to predict the ligand-binding sites. All the amino acid residues around the binding site (Supplementary Table 2) are marked to create a site for molecular docking.

### Molecular docking, rescoring and clustering analysis of docked poses

Docking was carried out using AutoDockVina in a virtual screening software, PyRx against the target proteins with the selected ligand database. The conformation with the lowest docked energy was chosen after the docking interactions since, the higher the negative binding energy value, the stronger is the binding of the ligand in the target [20].

The rescoring of docked poses was done by using the python implementation of NNScore 1.0 [21] in combination with a consensus of the top 24 scoring networks.

AuPosSOM (Automatic analysis of Poses using SOM) [22] was used for the clustering analysis and to differentiate active compounds from inactive ones. AuPosSOM default parameters were used. The tree was visualized using PhyloWidget [23].

### Protein-ligand interaction visualization

The 2D and 3D protein-ligand interaction for the lead compound was observed and analyzed using Discovery Studio Visualizer 2017 and ligplot+.

### ADME/Tox screening

The toxic profiles and drug-likeness based on the binding energies were predicted using the OSIRIS program [24]. OSIRIS calculates various drug relevant properties like molecular weight, cLogP, cLogS, Druglikeness, and toxicities like mutagenicity, tumorigenicity, reproductive effects and irritant effects in the lead molecules based on functional groups present in their structures [25].

## Results

### Drug target identification

**Fig. 1:**
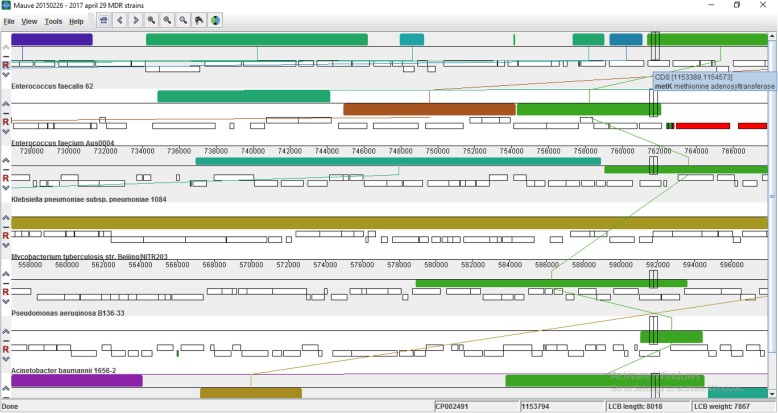
Multiple sequence alignment in MAUVE, showing MetK as a common gene in all 9 pathogen’s genomes aligned (data for remaining 5 not shown)

The MAUVE result showed MetK as one of the probable therapeutic targets and was taken as a reference on our search for other therapeutic targets (Fig. 1).

Manual curing of the alignment revealed twenty-four common genes found in most of the strains with diverse roles. Most of the genes (Supplementary Table 1) were ribosomal proteins (14 proteins) and some were involved in ATP synthesis (3 proteins), DNA directed RNA polymerase (2 proteins), chaperones (2 proteins), elongation factor (1 protein), protein translocator (1 protein), involved in thiol assimilation (1 protein) which were not pursued further due to lack of required computational resources to work on these.

### Amino acid alignment

**Fig. 2:**
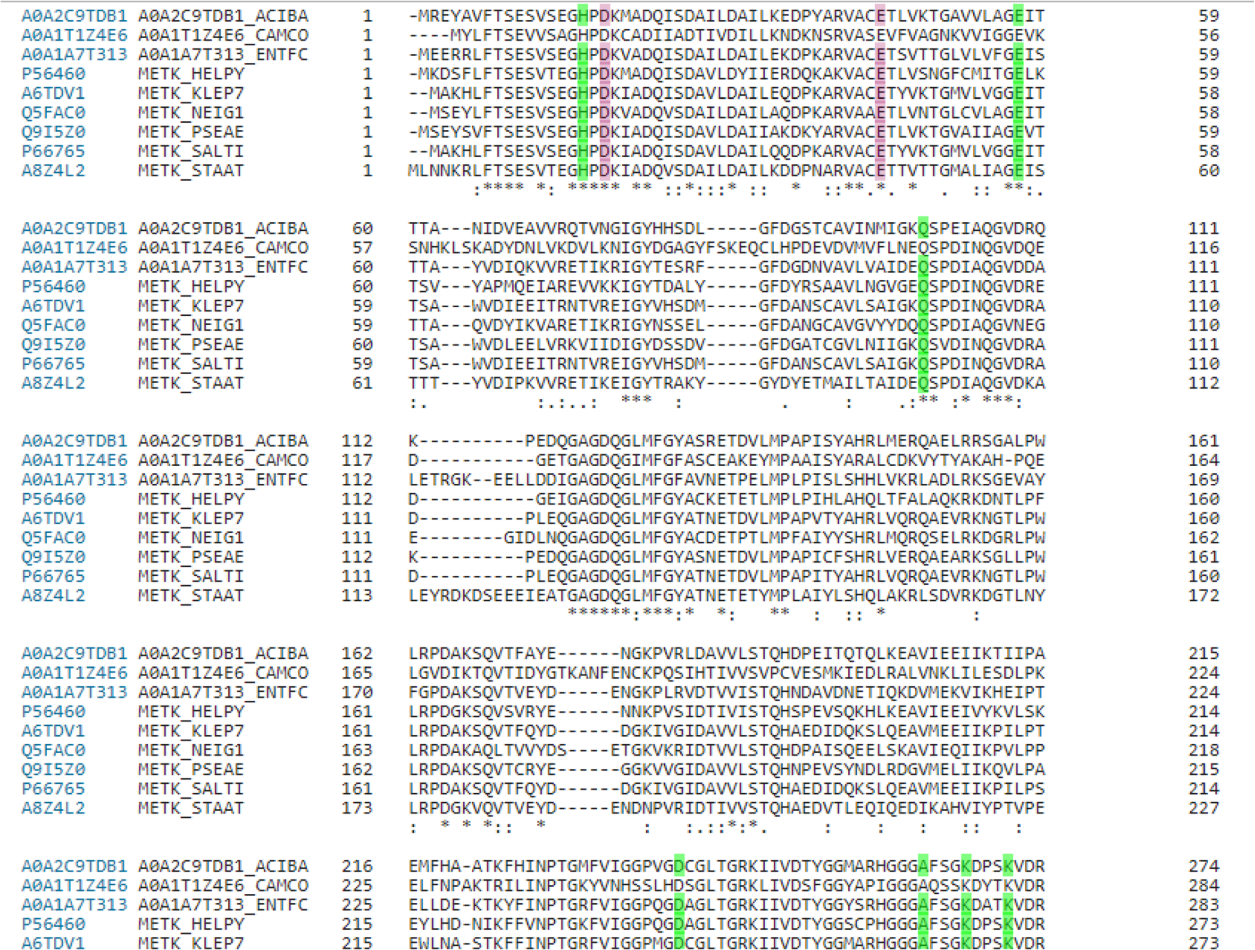
Clustal Omega alignment of MetK protein from eight pathogens

The active binding sites in MetK were found same (conserved) in all the pathogens under study upon amino acid sequence alignment using clustal Omega (Fig. 2) making this a common target in all these pathogens. Further analysis of gene essentiality for pathogen survival could reveal whether this is the lead target protein.

### Gene essentiality

Search in DEG and OGEE for gene essentiality of the genes under study in the target organisms revealed *metK* as essential in *H. pylori*, *P. aeruginosa*, *S. aureus* and *S. typhi*. Similarly, *dam* was found to be essential in *S. enterica* subsp. *enterica* serovar Typhimurium; *cobA* was reported as non-essential in *P. aeruginosa*; and *trmD* was essential in *P. aeruginosa, S. aureus, S. typhi* whereas non-essential in *H. pylori* and *Acinetobacter* sp. However, these databases do not make use of the interrelation between the genes to record gene essentiality. So the genes mentioned as non-essential here could still be essential when the activity of one is inhibited. Since our works were concerned with multiple targets simultaneously and all these genes under study are SAM utilizers, they were taken for further study despite being non-essential in some instances.

### Ligand database preparation

**Fig. 3:**
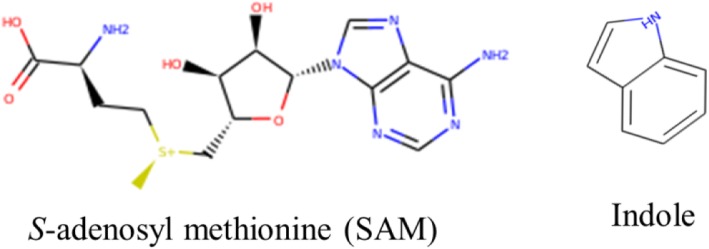
Chemical structures of SAM and Indole

**Fig. 4:**
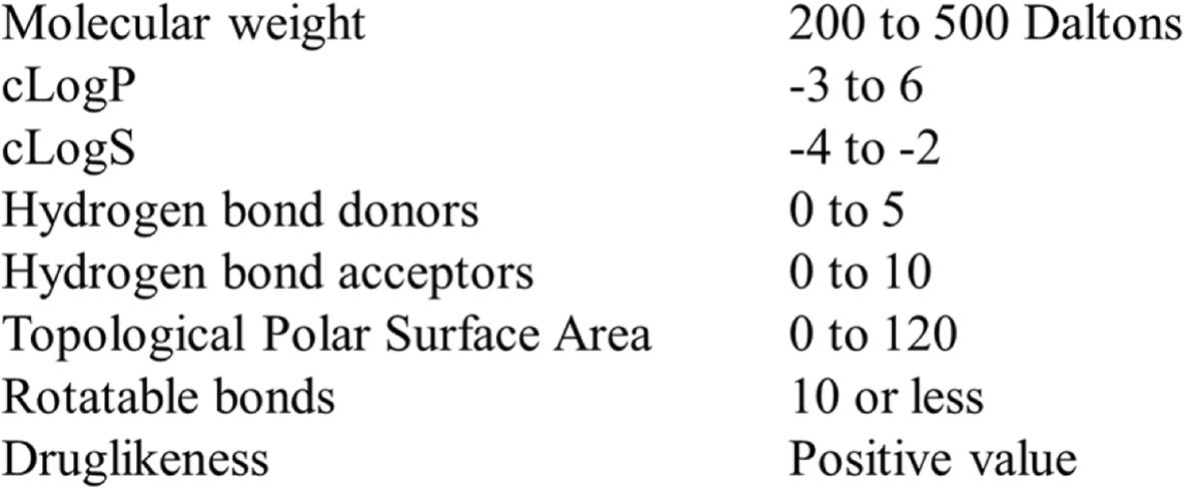
Drug-likeness parameters used for initial screening of ligands

The close structural proximity of the indole ring to the adenosyl moiety of SAM (Fig. 3) pushes indole derivatives to be probable candidates against SAM binding pocket of MetK. Thus, indole derivatives were presumed as the potential ligand sources for virtual screening. A total of 715 indole derivatives were taken from ZINC database among which only 102 showed the drug-like properties based on ADMET parameters (Fig. 4) and used for molecular docking studies.

### Molecular docking results

**Fig. 5:**
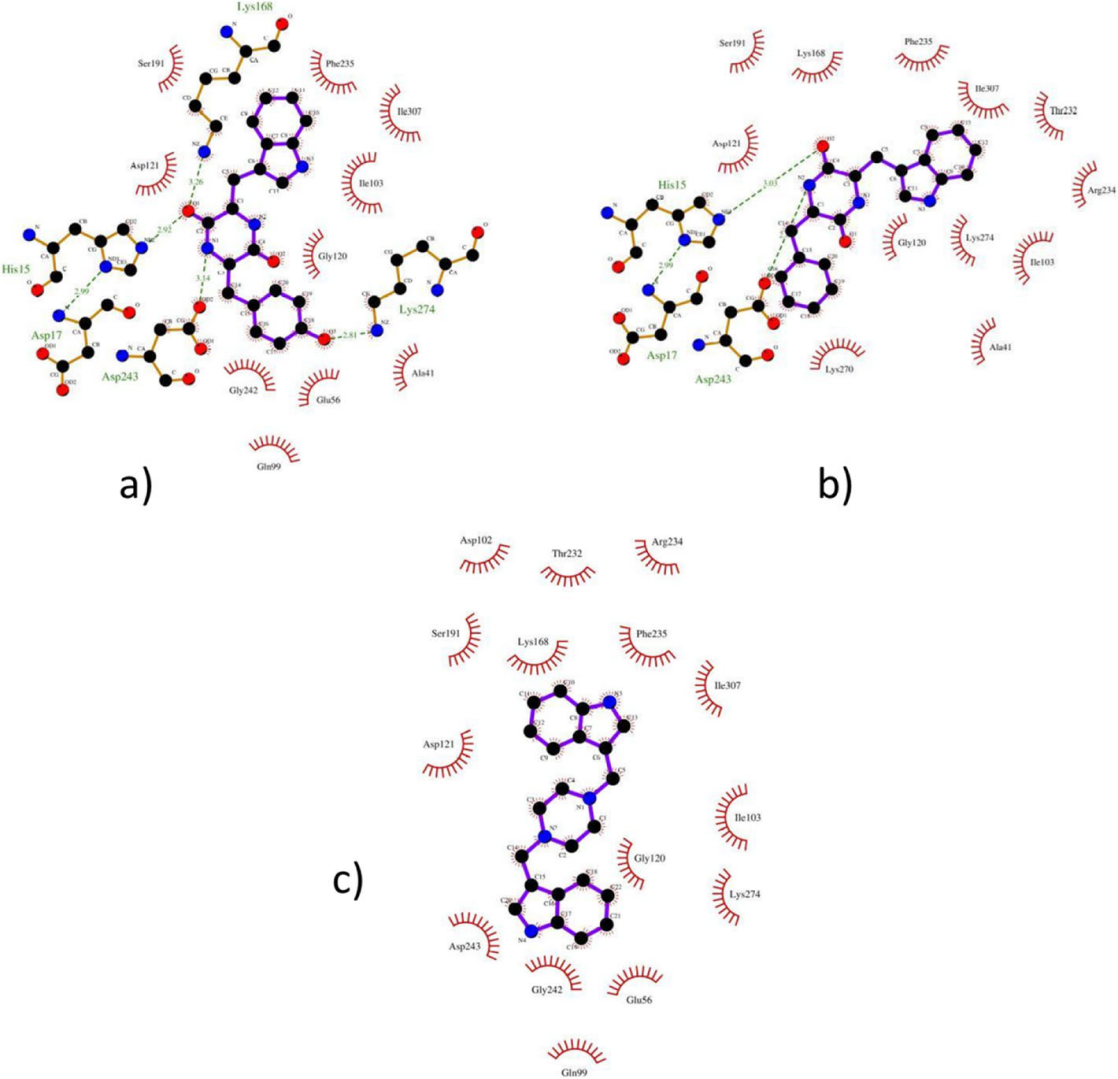
2D-interactions of **a**) ZINC04899565, **b**) ZINC06096559, and **c**) ZINC19909549 with amino acid residues at binding pocket of MetK of *N. gonorrhoeae*

One hundred two ligands that passed ADME/T tests were subjected to molecular docking against the MetK protein of *N. gonorrhoeae* (PDB id: 5T8S). Fifty three exhibited higher binding energy in the SAM binding pocket of MetK than its native ligand SAM (Supplementary Table 3). Top 3 ligands ZINC04899565 (B.E = − 11.0 Kcal/mol), ZINC06096559 (B.E = − 10.9 Kcal/mol) and ZINC19909549 (B.E = − 10.9 Kcal/mol) with higher B. E than that of SAM (B.E = − 8.7 Kcal/mol) were further investigated to look for their binding modes into the catalytic site of MetK. The common aminoacid residues involved for these three top ligands were Lys274, Gly120, Ile103, Ile307, Phe235, Lys168, Ser191, Asp121, and Asp243 which could have contributed in stronger binding affinities in the binding pocket of MetK (Fig. 5).

NNScore, a neural network based scoring function was then used to re-rank the small-molecule ligands which resulted in 12 positive hits (good binders) as potential inhibitors of MetK in *N. gonorrhoeae* (Supplementary Table 3). ZINC04899565 was still among the top binders.

**Fig. 6:**
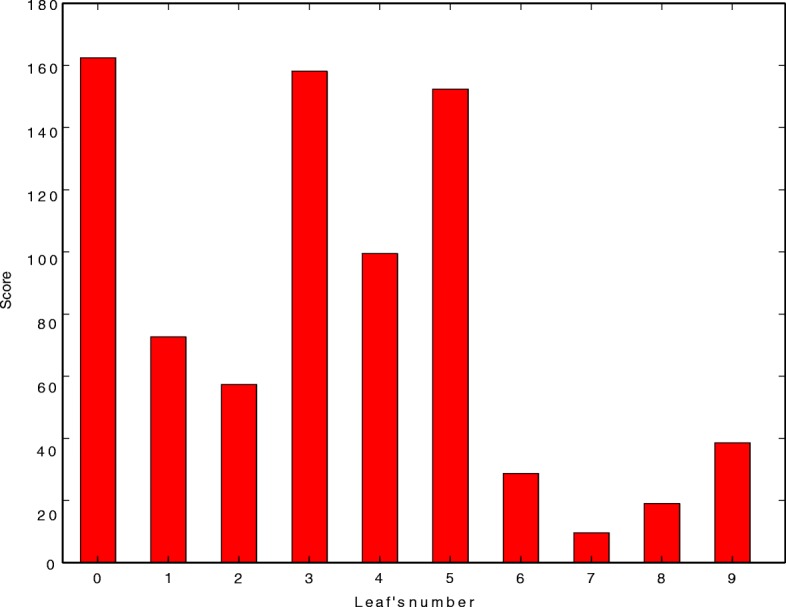
The scoring plot in AuPosSom

We further used a contact activity relationship (CAR) analysis to overcome the limitations of the scoring functions used for docking. Aupossom analyses all the docking poses in multiple conformations given by the docking algorithm and discriminates active and inactive compounds using only mean protein contacts’ footprints calculation. The 12 ligands along with SAM were clustered into 10 different groups with varied scores. The score is determined by the combination between contact specificity and contact intensity of the ligands with various atoms of the protein molecule. The plot (Fig. 6) shows the ligands in leaves 0, 3, 4 and 5 as the most active ones. ZINC01494627 from cluster 0, ZINC14824027 and ZINC49171024 from cluster 3, ZINC04899565 from cluster 4 and ZINC15219763 from cluster 5 can thus be concluded as the potential MetK inhibitors of *N. gonorrhoeae.*

**Fig. 7:**
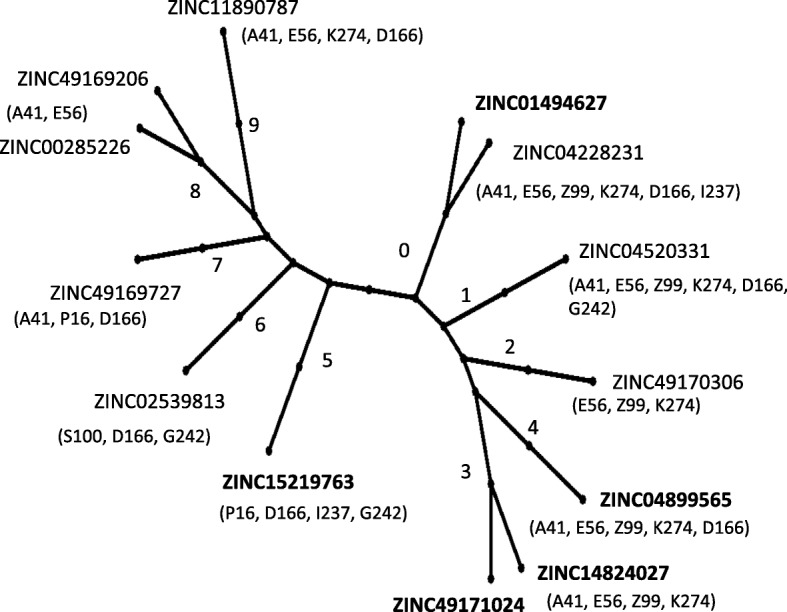
Tree representation of contact footprints clustering of 12 potential ligands and SAM for MetK. The clusters are numbered 0 to 9, each leaf representing a particular ligand. The contact footprints for each cluster are represented by a letter indicating an aminoacid followed by the position in the protein chain. Example, A41 refers to Alanine in the position 41. E, Z, S, K, P, D, I and G indicates Glutamic acid, Glutamine, Serine, Lysine, Proline, Aspartic acid, Isoleucine, and Glycine respectively

CAR results showed that these 12 potential leads were distributed in 10 different clusters (0 to 9) with different protein-contact footprints represented in the clustering tree (Fig. 7). The different clusters indicate the differences in the interacting residues with the protein. Cluster 0 contains 1 compound along with SAM (native ligand) which interacts predominantly with A41, E56, Z99, K274, D166 and I237 residues. Cluster 5 contains 1 compound that interacts predominantly with P16, D166, I237 and G242 residues and so on. The similar binding residues of the ligands with that of SAM could have contributed to their inhibition potential of MetK. CAR analysis can thus be used to cluster the compounds as per the binding residues of the protein.

Those 5 screened ligands on further docking against the SAM binding pocket of MetK of all other pathogens under study resulted in 3 potential leads (Table 1). These 3 lead molecules were further docked against all other protein targets CobA, Dam and TrmD (Tables 2, 3, 4) to assess their inhibition potential in multiple targets resulting 2 potential lead candidates (Table 5).

**Table 1:** Indole derivatives with MetK inhibition potential in all pathogens under study

Zinc Ids	*Ng* −8.7*	*Ab* −7.2*	*Cc* −6.4*	*Kp* − 6.2*	*Ef* −5.6*	*Hp* −4.7*	*Pa* − 6.0*	*Sa* − 6.1*	*St* − 6.1*
ZINC04899565	−10.4	−7.7	−8.3	− 7.4	−6.9	−5.3	− 6.9	−6.7	− 7.3
ZINC15219763	−9.5	−7.5	− 9.8	−8.3	− 7.1	− 6.1	−7.8	−6.9	− 7.5
ZINC49171024	−10.8	−7.6	− 7.9	−7.4	− 6.9	− 5.6	−7.8	− 6.9	−7.0

*Binding energy of native ligand

Ng: *Neisseria gonorrhoeae*; Ab: *Acinetobacter baumannii*; Cc: *Campylobacter coli*; Kp: *Klebsiella pneumoniae*; Ef: *Enterococcus faecium*; Hp: *Helicobacter pylori*; Pa: *Pseudomonas aeruginosa*; Sa: *Staphylococcus aureus*; St: *Salmonella typhi.*

**Table 2:** Indole derivatives with Dam inhibition potential in all pathogens under study

Zinc Ids	Ng − 6.8*	Ab −8.3*	Cc − 8.2*	Kp − 7.5*	Ef −6.6*	Hp − 8.0*	Pa	Sa − 8.7*	St − 8.6*
ZINC04899565	−7.7	− 9.2	− 9.5	− 9.4	− 6.9	−8.1	#	−9.6	− 9.5
ZINC49171024	−8.5	−9.5	− 10.1	−9.3	− 8.0	−8.9	#	−10.0	− 9.5

*Binding energy of native ligand

# B. E not calculated due to unavailability of valid 3D structure.

Ng: *Neisseria gonorrhoeae*; Ab: *Acinetobacter baumannii*; Cc: *Campylobacter coli*; Kp: *Klebsiella pneumoniae*; Ef: *Enterococcus faecium*; Hp: *Helicobacter pylori*; Pa: *Pseudomonas aeruginosa*; Sa: *Staphylococcus aureus*; St: *Salmonella typhi.*

**Table 3:** Indole derivatives with CobA inhibition potential in all pathogens under study

Zinc Ids	Ng − 6.7*	Ab − 8.8*	Cc − 7.9*	Kp − 8.4*	Ef	Hp − 7.7*	Pa − 6.0*	Sa − 7.4*	St −8.2*
ZINC04899565	−8.0	−9.5	−8.7	− 9.5	#	− 8.6	− 7.2	−8.4	−9.2
ZINC49171024	−7.3	−9.1	−9.2	− 9.2	#	−8.4	− 6.9	− 8.1	−9.2

*Binding energy of native ligand

# B. E not calculated due to unavailability of valid 3D structure.

**Table 4:** Indole derivatives with TrmD inhibition potential in all pathogens under study

Zinc Ids	Ng − 6.7*	Ab − 7.1*	Cc − 7.0*	Kp − 6.4*	Ef − 6.5*	Hp − 7.4*	Pa −8.1*	Sa − 7.7*	St − 7.0*
ZINC04899565	−7.3	− 7.9	− 7.6	− 8.0	−6.7	− 8.4	− 10.5	− 9.5	− 7.9
ZINC49171024	−7.7	− 8.1	−9.1	− 8.0	−8.1	− 8.9	− 9.7	−10.2	− 9.0

*Binding energy of native ligand

**Table 5:** Molecular properties of the lead candidates obtained from OSIRIS property explorer

Ligand names	Zinc Ids	MW	cLogP	cLogS	HBA	HBD	PSA	DL	nRotb
3-[(4-hydroxyphenyl)methyl]-6-(1H-indol-3-ylmethyl)piperazine-2,5-dione	ZINC04899565	349.4	1.5	−3.3	6	4	94.2	4.4	4
1-[(3S)-3-[5-(1H-indol-3-ylmethyl)-1,3,4-oxadiazol-2-yl]pyrrolidin-1-yl]ethanone	ZINC49171024	310.4	2.3	−2.4	6	1	75.0	5.2	3

MW: Molecular Weight; LogP: Logarithm of compound’s partition coefficient between n-octanol and water; LogS: Logarithm of a compound’s solubility measured in mol/liter; HBA: Number of hydrogen bond acceptors; HBD: Number of hydrogen bond donors; PSA: Topological Polar Surface Area; DL: Druglikeness score; nRotb: Number of rotatable bonds

### Cross-reactivity with human homologs

Questions on cross-reactivity with human homologs of S-adenosylmethionine synthase (Uniprot id: Q00266) could be raised as it has more than 50% structural similarity with that of bacteria (Table 6). Unfortunately, both leads were potential inhibitors of its human homolog as well (Supplementary Table 4).

**Table 6:** Alignment of metK of *Homo sapiens* and pathogens

Target organisms	% identity in human metK
*Neisseria gonorrhoeae*	66
*Salmonella typhi*	63
*Acinetobacter baumannii*	63
*Campylobacter coli*	41
*Enterococcus faecium*	58
*Helicobacter pylori*	62
*Klebsiella pneumoniae*	63
*Pseudomonas aeruginosa*	62
*Staphylococcus aureus*	59

Nevertheless, MetK inhibitors of humans could still be used as antibacterial therapeutics because of the presence of SAM transporters in humans [26] and the SAM requirements can be replenished from external sources. The lack of crystal structures of SAM transporters in humans constrained the molecular docking studies of possible inhibition of the transport system.

Human homologs for other target proteins were not considered because of their dissimilarity with humans.

## Discussion

### Drug target identification

Lead molecules with multiple target proteins in a single pathogen and with a common target in multiple pathogens are highly sought [27] to develop a broad-spectrum drug. This strategy has been designed for preventing easy resistance development against these new drugs. Developing resistance in multiple targets at once could be evolutionarily challenging for any pathogens and probably impossible for the bacteria to survive against such developed drugs. Hence, genome-level sequence alignment of major pathogens could give common new lead target proteins for the screening of lead inhibitor molecules of these proteins, MetK being the common target in this study (Fig. 1). From this, the respective metabolic pathway or other target proteins could be identified based on protein-protein interactions.

MetK codes the formation of SAM from ATP and methionine as substrates. SAM is utilized by three major metabolic pathways: transmethylation, transsulfuration, and polyamine synthesis making SAM an important molecule in normal cell functioning and survival [28]. In addition, SAM is a primary methyl donor in multiple reactions including corrin ring methylation [29], RNA methylation [30], and DNA methylation [31] thus, these steps were also taken as lead targets.

Quorum sensing is one of the major causes of resistance in pathogens that utilize autoinducers which inturn utilize SAM as a substrate [32]. Also, it controls biofilm formation and virulence in bacteria [33]. The literature search further verified the essentiality of MetK in many different pathogens [34, 35]. Also, the lack of reports about SAM transporters in any of the mentioned target pathogens makes this a better target. So, including MetK, other SAM utilizing proteins, namely CobA, Dam, and TrmD were taken as potential drug targets against which virtual screening of compounds was done.

### Indoles as potential drugs

The adenosyl moiety of SAM and ATP binding domain present in kinases [36] probably suggests kinase inhibitors as potential inhibitors of these proteins that biosynthesize or utilize SAM. Protein kinase inhibitors represent an important class of targeted therapeutic agents, particularly as anticancer drugs [37]. Several indoles have been reported to possess kinase inhibition potentials [38–41]. Also, Indole is reported to be metabolized by human cytochrome P450 2A6 [42] and the source of indole could be from tryptophan metabolism by gut microflora. This indicates that indole could be easily transported in humans through gut suggesting indoles are metabolized in humans thus indicating these could not pose toxicity [43]. In addition, indole has been suggested to be pharmaceutical scaffolds for drug development [44]. Also, its metabolized derivative Indirubin has been reported to be kinase inhibitor [45].

### ADMET screening

The primary reason for lead molecules not being able to pass the clinical trials is their inability to reach the target and perform its predicted function, and also the toxicity issues [46]. Thus, ADMET and pharmacokinetic properties evaluation in the early stages of drug discovery seem to be a wiser choice. The toxic profiles and drug-likeness were predicted by OSIRIS using various parameters. Various physicochemical and drug relevant properties such as Molecular weight, cLogP, cLogS, number of hydrogen bond donors and acceptors, topological polar surface area, number of rotatable bonds and drug-likeness were analyzed for each of the lead molecules. The parameters (Fig. 4) were set based on Lipinski’s rule of five which predicts whether a chemical compound has chemical and physical properties that would make it likely to be an orally active drug in humans. The rule states that most orally active drugs will have molecular weight ≤ 500, logP ≤5, hydrogen bond donors ≤5, and hydrogen bond acceptors ≤10. Also, the aqueous solubility of a compound measured as logS significantly affects its absorption and distribution characteristics. Low solubility usually goes along with a bad absorption so poorly soluble compounds should be avoided. The number of rotatable bonds determines the flexibility of compounds and can predict oral bioavailability. It has been reported that 10 or fewer rotatable bonds in a molecule indicated good oral bioavailability [47]. Polar surface area (PSA) of a molecule can predict membrane permeability including crossing of the blood-brain barrier. Most known drugs have PSA values less than 120 Å [48].

### Lead candidates

**Fig. 8:**
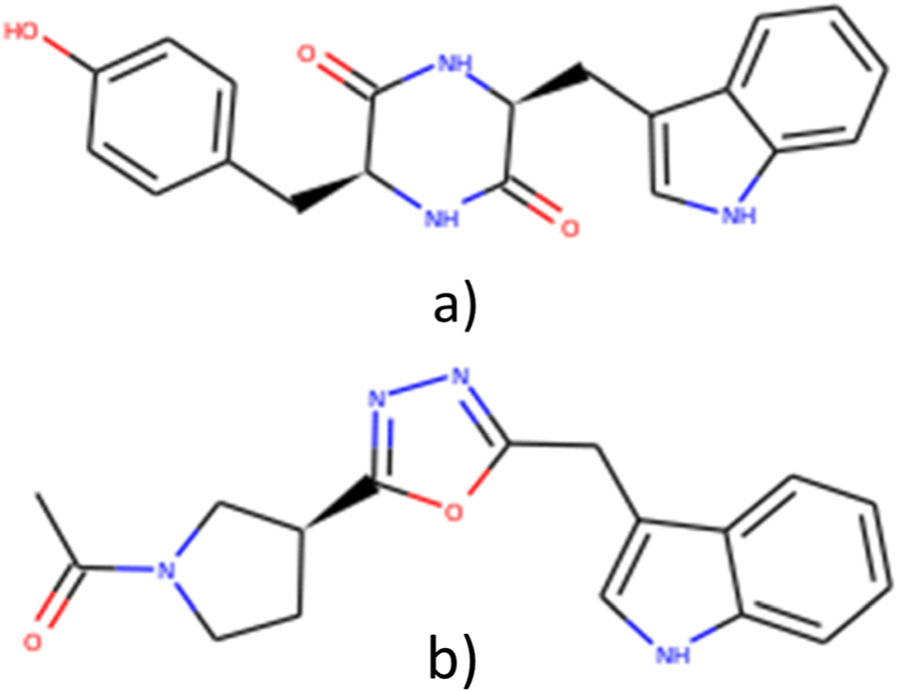
Potential lead candidates with potential broad-spectrum activities against the target pathogens under study **a**) ZINC04899565 **b**) ZINC49171024

ADMET evaluation of these two leads (Fig. 8) was done to access their possibility to be drug candidates. Both met the parameters for drug-likeness including Lipinski’s rule of five (Table 5).

Apart from indole, ZINC04899565 has a benzene and a 2,5-diketopiperazine ring. Out of all the naturally occurring peptide antibiotics, the 2,5-diketopiperazine rings are among the most numerous. Cycloserine diketopiperazine active against *Mycobacterium tuberculosis,* bicyclomycin active against gram-negative bacteria, avrainvillamide which contains a 3-alkylidene-3H-indole-1-oxide function active even against multi drug-resistant bacteria are some instances [49]. Moreover, 2,5-diketopiperazines have also been reported to inhibit quorum sensing in certain gram-negative bacteria thus blocking cell-to-cell communication and restraining the virulence as well [50]. Structurally, the 2,5-diketopiperazines are the smallest possible cyclic peptides, which are peptidoimmetic in nature resembling a constrained protein beta turn. They have two cis-amide bonds thus possessing 2H-bond acceptors and donors each. Although they contain conformationally constrained heterocyclic scaffolds, they are flexible since the six-membered ring can exist either in a flat or a slight puckered boat conformation. Moreover, these are stable to proteolysis [49]. All these features support them bind to a wide range of enzymes and receptors, and their good bioavailability and resistance to enzymatic degradation make them excellent drug candidates. Thus, ZINC04899565 has the potential to be a broad-spectrum antimicrobial based on this study and could be pursued further.

ZINC49171024 is an indole with a pyrrolidine and a benzimidazole ring. Pyrrolidine moiety containing compounds have been reported as antimicrobials and fungals [51]. The strong lipophilic properties of benzimidazoles contribute in producing antimicrobial effects [52]. All these features indicate the high probability of these two compounds to be developed as broad-spectrum antimicrobials.

## Conclusion

CADD approaches were used in this study to discover potential methyltransferase inhibitory activities of indole derivatives. Multiple protein targets were subjected to molecular docking that resulted ZINC04899565 and ZINC49171024 as probable therapeutic drug candidates with multi-target potential and probable antimicrobial resistance managers since these multiple targets would be troublesome for the pathogens to easily evolve resistance as these are really critical in its survival and mutating these targets could be more lethal than survival.

## Supplementary information

**Additional file 1: **Supplementary Table 1. Putative lead target proteins identified as common, by whole genome sequence alignment of WHO priority list pathogens [3] and *M. tuberculosis*.

**Additional file 2: **Supplementary Table 2. Predicted binding sites in MetK of *Helicobacter pylori* in 3DligandSite predicted via Phyre2 webserver.

**Additional file 3: **Supplementary Table 3. 53 indole derivatives with higher B.E in MetK of *Neisseria gonorrhoeae* than SAM with their NNScore values.

**Additional file 4:** Supplementary Table 4. Docking results of two lead molecules in human homolog of MetK, Mat2B (RCSB id: 4KTT).

## Data Availability

The 3D structures of the proteins and ligands used in the current study are available from RCSB PDB (https://www.rcsb.org/) and ZINC (http://zinc15.docking.org/) databases respectively. Molecular docking can be performed using PyRx (https://pyrx.sourceforge.io/). The library of screened ligands, 3D structures of homology modeled proteins and any other information regarding the current work will be made available by the corresponding author on reasonable request.

## Abbreviations

MDR: Multi Drug Resistant
WHO: World Health Organization
SAM: S-adenosyl Methionine
ADMET: Absorption, Digestion, Metabolism, Excretion and Toxicity
CADD: Computer Aided Drug Design
DEG: Database of Essential Genes
OGEE: Online GEne Essentiality database
CAR: Contact Activity Relationship

## References

[1] Tello A, Austin B, Telfer TC (2012). Selective pressure of antibiotic pollution on bacteria of importance to public health. Environ Health Perspect.

[2] Caniaux I, van Belkum A, Zambardi G, Poirel L, Gros MF. MCR: modern colistin resistance. Eur J Clin Microbiol Infect Dis, 2017;36:415–420. 10.1007/s10096-016-2846-y. 10.1007/s10096-016-2846-y 27873028

[3] University, T. Tufts CSDD assessment of cost to develop and win marketing approval for a new drug now published. In: Retrieved from development. Tufts: Center for the Study of Drug website; 2016. http://csdd.tufts.edu/news/complete_story/tufts_csdd_rd_cost_study_now_published.

[4] Sliwoski G, Kothiwale S, Meiler J, Lowe EW (2014). Computational methods in drug discovery. Pharmacol Rev.

[5] Lawe-Davies O, Bennett S. WHO publishes list of bacteria for which new antibiotics are urgently needed: WHO; 2017. p. 1. Retrieved from http://www.who.int/mediacentre/news/releases/2017/bacteria-antibiotics-needed/en/.

[6] Darling ACE, Mau B, Blattner FR, Perna NT (2004). Mauve: multiple alignment of conserved genomic sequence with rearrangements. Genome Res.

[7] Park JY, Kim S, Kim SM, Cha SH, Lim SK, Kim J (2011). Complete genome sequence of multidrug-resistant *Acinetobacter baumannii* strain 1656-2 Which Forms Sturdy Biofilm. J Bacteriology.

[8] Pearson BM, Rokney A, Crossman LC, Miller WG, Wain J, van Vliet AHM (2013). Complete genome sequence of the *Campylobacter coli* clinical isolate 15-537360. Genome Announcements.

[9] Lam MMC, Seemann T, Bulach DM, Gladman SL, Chen H, Haring V, Stineara TP (2012). Comparative analysis of the first complete *Enterococcus faecium* genome. J Bacteriol.

[10] Avasthi TS, Devi SH, Taylor TD, Kumar N, Baddam R, Kondo S, Ahmed N (2011). Genomes of two chronological isolates (*Helicobacter pylori* 2017 and 2018) of the west African *Helicobacter pylori* strain 908 obtained from a single patient. J Bacteriol.

[11] Lin AC, Liao TL, Lin YC, Lai YC, Lu MC, Chen YT (2012). Complete genome sequence of *Klebsiella pneumoniae* 1084, a hypermucoviscosity-negative K1 clinical strain. J Bacteriol.

[12] Silva C, Calva E, Puente JL, Zaidi MB, Vinuesa P (2016). Complete genome sequence of *Salmonella enterica* Serovar Typhimurium strain YU15 (sequence type 19) harboring the Salmonella Genomic Island 1 and virulence plasmid pSTV. Genome Announcements.

[13] Dranow DM, Delker SL, Lorimer DD, Edwards TE. Crystal structure of a S-adenosylmethionine synthase from *Neisseria gonorrhoeae* with bound S-adenosylmethionine. AMP, Pyrophosphate, Phosphate, and Magnesium. n.d.. 10.2210/PDB5T8S/PDB.

[14] Storbeck, S, Saha, S, Krausze, J, Klink, BU, Heinz, DW, Layer, G. (2011). Crystal structure of the heme d 1 biosynthesis enzyme NirE in complex with its substrate reveals new insights into the catalytic mechanism of S-adenosyl-L-methionine-dependent uroporphyrinogen III methyltransferases. J Biol Chem 10.1074/jbc. M111.239855. 10.1074/jbc.M111.239855 PMC3143637 21632530

[15] Jaroensuk, J, Wong, YH, Zhong, W, Liew, CW, Maenpuen, S, Sahili, AE, Fuangthong, M. (2019). Crystal structure and catalytic mechanism of the essential m 1 G37 tRNA methyltransferase TrmD from *Pseudomonas aeruginosa.*10.1261/rna.066746.118. 10.1261/rna.066746.118 PMC6795141 31399541

[16] Netz N, Opatz T (2015). Marine indole alkaloids. Marine Drugs.

[17] Irwin JJ, Shoichet BK (2005). ZINC - a free database of commercially available compounds for virtual screening. J Chem Inf Model.

[18] O’Boyle NM, Banck M, James CA, Morley C, Vandermeersch T, Hutchison GR. Open babel: an open chemical toolbox. Journal of Cheminformatics. 2011;3(10). 10.1186/1758-2946-3-33. 10.1186/1758-2946-3-33 PMC3198950 21982300

[19] Wass MN, Kelley LA, Sternberg MJE. 3DLigandSite: predicting ligand-binding sites using similar structures. Nucleic Acids Res. 2010;38(SUPPL. 2). 10.1093/nar/gkq406. 10.1093/nar/gkq406 PMC2896164 20513649

[20] Dallakyan S, Olson AJ (2015). Small-molecule library screening by docking with PyRx. Methods Mol Biol.

[21] Durrant JD, McCammon JA. NNScore: a neural-network-based scoring function for the characterization of protein-ligand complexes. J Chem Inf Model. 2010. 10.1021/ci100244v. 10.1021/ci100244v PMC2964041 20845954

[22] Bouvier G, Evrard-todeschi N, Girault JP, Bertho G. Automatic clustering of docking poses in virtual screening process using self-organizing map. Bioinformatics. 2009. 10.1093/bioinformatics/btp623. 10.1093/bioinformatics/btp623 19910307

[23] Jordan GE, Piel WH. PhyloWidget: web-based visualizations for the tree of life. Bioinformatics. 2008. 10.1093/bioinformatics/btn235. 10.1093/bioinformatics/btn235 18487241

[24] Nisha CM, Kumar A, Nair P, Gupta N, Silakari C, Tripathi T, Kumar A. Molecular docking and in silico admet study reveals acylguanidine 7a as a potential inhibitor of β -secretase. Adv Bioinforma. 2016;2016. 10.1155/2016/9258578. 10.1155/2016/9258578 PMC4842033 27190510

[25] Sander T, Freyss J, Von Korff M, Rufener C (2015). DataWarrior: an open-source program for chemistry aware data visualization and analysis. J Chem Inf Model.

[26] Agrimi G, Di Noia MA, Marobbio CMT, Fiermonte G, Lasorsa FM, Palmieri F (2004). Identification of the human mitochondrial S-adenosylmethionine transporter: bacterial expression, reconstitution, functional characterization and tissue distribution. Biochem J.

[27] Bolognesi L, M. (2013). Polypharmacology in a single drug: multitarget drugs. Curr Med Chem.

[28] Lu, S. C. (2000). S-Adenosylmethionine. Int J Biochem Cell Biol, 32(4), 391–395. 10.1016/S1357-2725(99)00139-9. 10.1016/s1357-2725(99)00139-9 10762064

[29] Roper, J. M., Raux, E., Brindley, A. A., Schubert, H. L., Gharbia, S. E., Shah, H. N., & Warren, M. J. (2000). The enigma of cobalamin (vitamin B12) biosynthesis in *Porphyromonas gingivalis*: identification and characterization of a functional corrin pathway. J Biol Chem, 275(51), 40316–40323. 10.1074/jbc. M007146200. 10.1074/jbc.M007146200 11007789

[30] Motorin Y, Helm M (2011). RNA nucleotide methylation. Wiley Interdisciplinary Reviews: RNA.

[31] Sánchez-Romero MA, Cota I, Casadesús J (2015). DNA methylation in bacteria: from the methyl group to the methylome. Curr Opin Microbiol.

[32] Wei Y, Perez LJ, Ng WL, Semmelhack MF, Bassler BL. Mechanism of *Vibrio cholerae* autoinducer-1 biosynthesis. ACS Chem Biol. 2011;6(4):356–65. 10.1021/cb1003652. 10.1021/cb1003652 PMC3077805 21197957

[33] Rutherford ST, Bassler BL. Bacterial quorum sensing: its role in virulence and possibilities for its control. *Cold Spring Harbor Perspectives in Medicine*, Vol. 2012;2. 10.1101/cshperspect.a012427. 10.1101/cshperspect.a012427 PMC3543102 23125205

[34] Walker DJF, Heap JT, Winzer K, Minton NP (2016). A genetic assay for gene essentiality in *Clostridium*. Anaerobe.

[35] Wei Y, Newman EB. Studies on the role of the metK gene product of *Escherichia coli* K-12. Mol Microbiol. 2002;43(6):1651–6. 10.1046/j.1365-2958.2002.02856.x. 10.1046/j.1365-2958.2002.02856.x 11952912

[36] Villamor, J G, Kaschani, F, Colby, T, Oeljeklaus, J, Zhao, D, Kaiser, M, van der Hoorn, RAL (2013). Profiling protein kinases and other ATP binding proteins in *Arabidopsis* using acyl-ATP probes. Mol Cell Proteomics, 12(9), 2481–2496. 10.1074/mcp. M112.026278. 10.1074/mcp.M112.026278 PMC3769325 23722185

[37] Grant SK (2009). Therapeutic protein kinase inhibitors. Cell Mol Life Sci.

[38] Chowdhury S, Sessions EH, Pocas JR, Grant W, Schröter T, Lin L, Feng Y. Discovery and optimization of indoles and 7-azaindoles as rho kinase (ROCK) inhibitors (part-I). Bioorganic and Medicinal Chemistry Letters. 2011. 10.1016/j.bmcl.2011.09.083. 10.1016/j.bmcl.2011.09.083 22004718

[39] Kiliç Z, Işgör YG, Olgen S (2009). Evaluation of new indole and bromoindole derivatives as pp60(c-Src) tyrosine kinase inhibitors. Chem Biol Drug Des.

[40] Ölgen S, Kiliç-Kurt Z, Şener F, Işgör YG, Çoban T (2011). Evaluation of novel aminomethyl indole derivatives as Src kinase inhibitors and antioxidant agents. Chemotherapy.

[41] Polychronopoulos P, Magiatis P, Skaltsounis AL, Myrianthopoulos V, Mikros E, Tarricone A, Meijer L. Structural basis for the synthesis of Indirubins as potent and selective inhibitors of glycogen synthase Kinase-3 and Cyclin-dependent kinases. J Med Chem. 2004. 10.1021/jm031016d. 10.1021/jm031016d 14761195

[42] Gillam EMJ, Notley LM, Cai H, De Voss JJ, Guengerich FP (2000). Oxidation of indole by cytochrome P450 enzymes. Biochemistry.

[43] Banoglu E, Jha GG, King RS (2001). Hepatic microsomal metabolism of indole to indoxyl, a precursor of indoxyl sulfate. Eur J Drug Metab Pharmacokinet.

[44] Suenkel B, Fischer F, Steegborn C (2013). Inhibition of the human deacylase Sirtuin 5 by the indole GW5074. Bioorganic and Medicinal Chemistry Letters.

[45] Wu ZL, Aryal P, Lozach O, Meijer L, Guengerich FP (2005). Biosynthesis of new indigoid inhibitors of protein kinases using recombinant cytochrome P450 2A6. Chem Biodivers.

[46] Hughes JP, Rees S, Kalindjian SB, Philpott KL (2011). Principles of early drug discovery. Br J Pharmacol.

[47] Veber DF, Johnson SR, Cheng HY, Smith BR, Ward KW, Kopple KD. Molecular properties that influence the oral bioavailability of drug candidates. J Med Chem. 2002. 10.1021/jm020017n. 10.1021/jm020017n 12036371

[48] Ertl P, Rohde B, Selzer P. Fast calculation of molecular polar surface area as a sum of fragment-based contributions and its application to the prediction of drug transport properties. J Med Chem. 2000. 10.1021/jm000942e. 10.1021/jm000942e 11020286

[49] Borthwick AD. 2,5-diketopiperazines: synthesis, reactions, medicinal chemistry, and bioactive natural products. Chem Rev. 2012. 10.1021/cr200398y. 10.1021/cr200398y 22575049

[50] Scoffone VC, Chiarelli LR, Makarov V, Brackman G, Israyilova A, Azzalin A, Buroni S. Discovery of new diketopiperazines inhibiting *Burkholderia cenocepacia* quorum sensing *in vitro* and *in vivo*. Sci Rep. 2016. 10.1038/srep32487. 10.1038/srep32487 PMC5007513 27580679

[51] Muneer S, Memon S, Pahnwar QK, Bhatti AA, Khokhar TS. Synthesis and investigation of antimicrobial properties of pyrrolidine appended calix [4] arene. Journal of Analytical Science and Technology. 2017. 10.1186/s40543-017-0111-3.

[52] Alasmary FAS, Snelling AM, Zain ME, Alafeefy AM, Awaad AS, Karodia N. Synthesis and evaluation of selected benzimidazole derivatives as potential antimicrobial agents. Molecules. 2015. 10.3390/molecules200815206. 10.3390/molecules200815206 PMC6332381 26307956

