# Supplementary material for: Prospects of Indole derivatives as methyl transfer inhibitors: antimicrobial resistance managers
Source: BMC Pharmacol Toxicol. 2020 May 4;21:33. doi: 10.1186/s40360-020-00402-9 (PMC7197119; doi:10.1186/s40360-020-00402-9)
Supplement: Supplementary file 1 — Additional file 1: Supplementary Table 1. Putative lead target proteins identified as common, by whole genome sequence alignment of WHO priority list pathogens [3] and M. tuberculosis. [file 40360_2020_402_MOESM1_ESM.docx]

Supplementary Table 1: Putative lead target proteins identified as common, by whole genome sequence alignment of WHO priority list pathogens (Lawe-Davies and Bennett, 2017) and M. tuberculosis.

| **Protein type** | **Gene: Protein Name (Reference)** |
| --- | --- |
| Ribosomal proteins (30s)  Ribosomal proteins (50s) | *rps*C: 30s ribosomal protein subunit S3 (PMID: P0A7V3); *rps*E: ribosomal protein S5 (PMID: P0A7W1); *rps*H: 30s ribosomal protein S8 (PMID: P0A7W7); *rps*N: 30s ribosomal protein S14 (PMID: P0AG59); *rps*Q: 30s ribosomal protein subunit S17 (PMID: P0AG63);  *rpl*C: 50s ribosomal protein L5 (PMID: 6760192); *rpl*F: 50s ribosomal protein L6 (PMID: P0AG55) ; *rpl*N: ribosomal protein L14(PMID: P0ADY3); *rpl*O: 50s ribosomal protein L15 (PMID: 3298242) but not aligned in *M. tuberculosis*; *rpl*P: 50s ribosomal protein subunit L16 (PubMed: 8524654); *rpl*R: 50s ribosomal protein L18 (PMID: 354687); *rpl*X: ribosomal protein L24 (PMID: 357435); *rpm*C: 50s ribosomal protein subunit L29 (PMC 327435); *rpm*D: 50s ribosomal protein L30 (Pubmed 3297162); |
| Involved in ATP synthesis | *atp*A: ATP synthase subunit alpha (EC:3.6.3.14); *atp*G: ATP synthase gamma chain (Uniprot ID: P95788); *atp*D: ATP synthase subunit beta (EC:3.6.3.14). |
| DNA directed RNA polymerase | *rpo*B: DNA-directed RNA polymerase, beta subunit (PMID: 1646077); *rpo*C: DNA-directed RNA polymerase, beta subunit (PMID: 1646077). |
| Chaperone (absent in *M. tuberculosis*) | *Dna*k: chaperone protein Dnak (Uniprot ID: P9WMJ9); *clp*B: chaperone protein ClpB (PMC1819559, PMID:12624113, PMC1821349). |
| Elongation factor | *fus*A: Elongation factor G (Uniprot ID: P0A6M8) |
| Protein translocator | *sec*Y: Protein translocase subunit (Pubmed 1531482, Pubmed 7650029). |
| Thiol assimilation | *MetK*: methionine adenosyl transferase (PMID: 6251075). |
