# Supplementary material for: Prospects of Indole derivatives as methyl transfer inhibitors: antimicrobial resistance managers
Source: BMC Pharmacol Toxicol. 2020 May 4;21:33. doi: 10.1186/s40360-020-00402-9 (PMC7197119; doi:10.1186/s40360-020-00402-9)

Supplementary Table 2: Predicted binding sites in MetK of Helicobacter pylori in 3DligandSite predicted via Phyre2 webserver


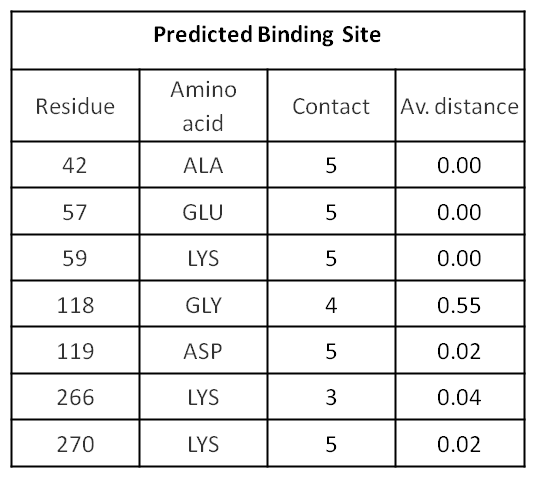

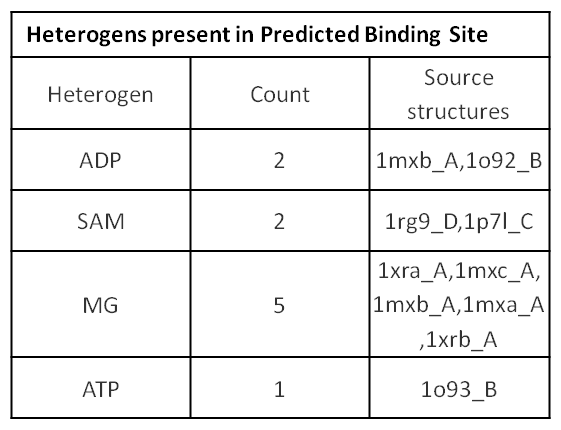

Supplement: Supplementary file 2 — Additional file 2: Supplementary Table 2. Predicted binding sites in MetK of Helicobacter pylori in 3DligandSite predicted via Phyre2 webserver. [file 40360_2020_402_MOESM2_ESM.docx]
