# Supplementary material for: Prospects of Indole derivatives as methyl transfer inhibitors: antimicrobial resistance managers
Source: BMC Pharmacol Toxicol. 2020 May 4;21:33. doi: 10.1186/s40360-020-00402-9 (PMC7197119; doi:10.1186/s40360-020-00402-9)
Supplement: Supplementary file 3 — Additional file 3: Supplementary Table 3. 53 indole derivatives with higher B.E in MetK of Neisseria gonorrhoeae than SAM with their NNScore values. [file 40360_2020_402_MOESM3_ESM.docx]

Supplementary table 3: 53 indole derivatives with higher B.E in metK of *Neisseria gonorrhoeae* than SAM with their NNScore values

| Ligands | B.E in metK (kcal/mol) | NNScore |
| --- | --- | --- |
| ZINC04899565 | -11 | 0.735902205 |
| ZINC06096559 | -10.9 | 0.417973444 |
| ZINC19909549 | -10.9 | 0.155534883 |
| ZINC45256267 | -10.9 | -0.180981163 |
| ZINC04899716 | -10.8 | 0.259975906 |
| ZINC13369641 | -10.6 | 0.554001816 |
| ZINC49171024 | -10.5 | 0.638996453 |
| ZINC04520331 | -10.2 | 0.743168982 |
| ZINC01494627 | -10.2 | 0.694111897 |
| ZINC02627687 | -10.2 | 0.688840217 |
| ZINC49169056 | -10.2 | 0.644843283 |
| ZINC49169206 | -10.2 | -0.01000185 |
| ZINC49169727 | -10.2 | -0.126841794 |
| ZINC06096622 | -10.1 | 0.583270223 |
| ZINC13375360 | -10.1 | 0.388367606 |
| ZINC28971613 | -10.1 | -0.062508323 |
| ZINC00285226 | -10 | 0.638717565 |
| ZINC04163159 | -10 | 0.613133553 |
| ZINC49170306 | -10 | 0.413042297 |
| ZINC49171033 | -10 | 0.384977635 |
| ZINC03230765 | -9.9 | -0.416060734 |
| ZINC04520329 | -9.8 | 0.585574018 |
| ZINC01682922 | -9.8 | 0.417695755 |
| ZINC03780014 | -9.8 | 0.205193625 |
| ZINC04163163 | -9.8 | -0.13263522 |
| ZINC45256608 | -9.8 | -0.48277648 |
| ZINC96113202 | -9.8 | -0.710350083 |
| ZINC58535134 | -9.7 | 0.832279505 |
| ZINC11890783 | -9.7 | 0.662160269 |
| ZINC14824027 | -9.7 | -0.126958781 |
| ZINC15219763 | -9.7 | -0.910417358 |
| ZINC53151307 | -9.6 | -0.141131488 |
| ZINC00128785 | -9.5 | 0.535609171 |
| ZINC00298330 | -9.4 | 0.303250384 |
| ZINC12401855 | -9.4 | -0.125322992 |
| ZINC13677763 | -9.4 | -0.611233356 |
| ZINC00298332 | -9.3 | 0.256709112 |
| ZINC14724327 | -9.2 | -0.129855871 |
| ZINC00014457 | -9.1 | 0.831702106 |
| ZINC04498255 | -9.1 | 0.445685935 |
| ZINC11890787 | -9.1 | 0.436747704 |
| ZINC12401856 | -9.1 | 0.282327015 |
| ZINC02539813 | -9 | 0.837509766 |
| ZINC02560262 | -9 | -0.009126236 |
| ZINC05600522 | -9 | -0.158857994 |
| ZINC12401854 | -9 | -0.361765579 |
| ZINC12401857 | -9 | -0.958202246 |
| ZINC00566973 | -8.9 | 0.536547335 |
| ZINC02466097 | -8.9 | 0.355194153 |
| ZINC04240327 | -8.9 | 0.087738792 |
| ZINC12371832 | -8.9 | 0.084379337 |
| ZINC12371833 | -8.9 | 0.025898034 |
| ZINC15120069 | -8.8 | -0.161117672 |

- The negative NNScore values indicate bad binders and the positive values include good binders, higher values indicate better binding.
