# Supplementary material for: Prospects of Indole derivatives as methyl transfer inhibitors: antimicrobial resistance managers
Source: BMC Pharmacol Toxicol. 2020 May 4;21:33. doi: 10.1186/s40360-020-00402-9 (PMC7197119; doi:10.1186/s40360-020-00402-9)
Supplement: Supplementary file 4 — Additional file 4: Supplementary Table 4. Docking results of two lead molecules in human homolog of MetK, Mat2B (RCSB id: 4KTT). [file 40360_2020_402_MOESM4_ESM.docx]

Supplementary Table 4: Docking results of two lead molecules in human homolog of MetK, Mat2B (RCSB id: 4KTT)

| Ligands | Binding Energy (Kcal/mol)  -8.7* |
| --- | --- |
| ZINC04899565 | -10.8 |
| ZINC49171024 | -10.1 |
|  |  |

- Binding energy of native ligand
